# Expression of aryl hydrocarbon receptor in rat brain lesions following traumatic brain injury

**DOI:** 10.1186/s13000-016-0522-2

**Published:** 2016-08-09

**Authors:** Kai Xu, Zicheng Yang, Rongchen Shi, Chunxia Luo, Zhiren Zhang

**Affiliations:** 1 Institute of Immunology, Third Military Medical University of PLA, 30 Gaotanyan Main Street, Chongqing, 400038 People’s Republic of China; 2 Department of Neurology, Southwest Hospital, Third Military Medical University, Chongqing, 400038 China

**Keywords:** Traumatic brain injury, T lymphocytes, Aryl hydrocarbon receptor

## Abstract

**Background:**

Aryl Hydrocarbon Receptor (AhR) is a ligand-activated transcription factor with multiple functions operating in a variety of organs, including the brain. Recent studies have revealed that AhR played a functional role in traumatic injuries. This paper aims to study the expression of AhR during the early phase following a traumatic brain injury (TBI) in rat brains by immunohistochemistry.

**Methods:**

Weight-drop induced TBI was performed in rats. The expression of AhR in brain of TBI rats were examined by immunohistochemistry.

**Results:**

Neuron expression of AhR in the rat brains of experiment group had been upregulated since day 3 in lesional hemisphere compared to that of the control group and mainly located in the cytoplasm, indicating an inactivated state. Interestingly, the accumulation of AhR^+^ non-neuron cells became significant as early as 18 h after injury, which had kept increasing until 24 h post injury and then decreased slowly. For AhR^+^ non-neuron cells, the AhR mainly located in cell nucleus, indicating a reactive status. Furthermore, double staining showed that most AhR^+^ non-neuron cells co-localized with W3/13, a marker for T lymphocytes, but not with ED-1 (for activated microglia/macrophages) or GFAP (for activated astrocytes), suggesting that most AhR^+^ non-neuron cells were T lymphocytes.

**Conclusion:**

This is the first study concerning AhR expression in brains following TBI, and our data demonstrated that AhR was upregulated and activated in T lymphocytes following TBI. More research is needed to make a more conclusive conclusion.

## Background

Traumatic brain injury (TBI) is a major public health threat in the society. While the contact and initial force cause multifocal lesions, the secondary injury can be more dreadful and diffuse. Delayed neuronal death in secondary injury can be due to several cellular and subcellular cascades such as release of excitatory amino acid (EAA), influx of Ca^++^_,_ and mechanical distortion of the cell membrane [1].

Immune responses in the CNS have recently drawn lots of attention, despite its long-lasting perception as a site of immune privilege [2]. The infiltration of immune cells following TBI has been well documented for some cell types, including neutrophils, microglia/macrophages and T lymphocytes [3]. Activated neutrophils upregulate adhesion molecules to enhance their association and migration into tissues and produce reactive intermediates, cytokines and chemokines, etc. Activated microglia/macrophages show high heterogeneity, and the activation may be beneficial, deleterious, or neutral [2]. Moreover, functions of T lymphocytes are more complicated. Both CD4^+^ and CD8^+^ T cells that enter the brain [4] and different subsets of T cells are involved in contradictory activities like a double-edged sword [5]. These immune cells orchestrate and lead to neuronal degeneration or regeneration/repair depending on the context [2]. However, details on the changes and exact roles of immune cells following TBI remain scarce.

Aryl Hydrocarbon Receptor (AhR) is a ligand-activated transcription factor of the basic helix-loop-helix/per-ARNT-Sim (bHLH/PAS) superfamily [6]. It can be activated by a variety of exogenous and endogenous compounds [7]. Upon ligand binding, AhR translocates into the nucleus to form an active heterodimeric complex [8] and triggers rapid transcriptional activations [9] in a variety of organs and cell systems. In addition to the classical knowledge of responding to environmental toxicants via exogenous ligands, recent studies pointed out that the physiological functions of AhR included cell differentiation, apoptosis [10, 11], immune response, [12] and injury repairing [13, 14].

AhR was identified in the brain [15], regulating neuronal cell cycle control and apoptosis [16]. Moreover, AhR has been identified in T lymphocytes and macrophages as a regulator of cytokine production [17]. Recent studies also revealed that AhR is involved in controlling helper T cell differentiation [18, 19]. However, the expression of AhR following TBI remains unknown. Therefore, this experiment was performed to study the spatiotemporal expression of AhR in brains of weight-drop induced TBI rats.

## Methods

### TBI brain tissue library

The brain libraries of the control group and the experiment group of rats used in this study have been described previously [20]. In brief, weight-drop induced TBI was performed in Lewis rats. This study was approved by the Ethics Committee of Tuebingen University (No. HF 2/06).

### Immunohistochemistry

A previous reported protocol for immunohistochemistry was applied to detect the expression of different proteins using following primary antibodies: anti-AhR (1:100; Lifespan Bioscience, Seattle, USA), anti-ED-1 (1:100; Serotec, Oxford, Great Britain) for macrophages, anti-W3/13 (1:100; Serotec, Oxford, Great Britain) for T lymphocytes and anti-glial fibrillary acidic protein for astrocytes (GFAP; 1:500; Chemicon International, Temecula, CA, USA) [20, 21].

### Data acquisition and statistical analysis

After immunostaining, brain sections were examined by light microscopy. For single AhR staining, the numbers of AhR^+^ cells were counted in 8 non-overlapping high-power fields (HPFs, x400 magnification) for each section. Results were presented as arithmetic means of AhR^+^ cells per HPF and standard errors of means (SEM). Statistical analysis was performed by one-way ANOVA followed by Dunnett’s multiple comparison tests (Graph Pad Prism 4.0 software). The significance levels were set at *p* < 0.05.

## Results

In this study, open skull weight drop injury generates a reproducible local lesion in the ipsilateral cortex. The development of the lesion was first analyzed by Hematoxylin and Eosin (HE) Staining and has been described previously by Zhang [20]. In brief, significant leukocyte infiltration and hemorrhage can be observed at the first day after injury. At day 4 post injury, a lesioned cavity together with its surrounding perilesional tissue loss formed under the impact areas.

**Fig. 1:**
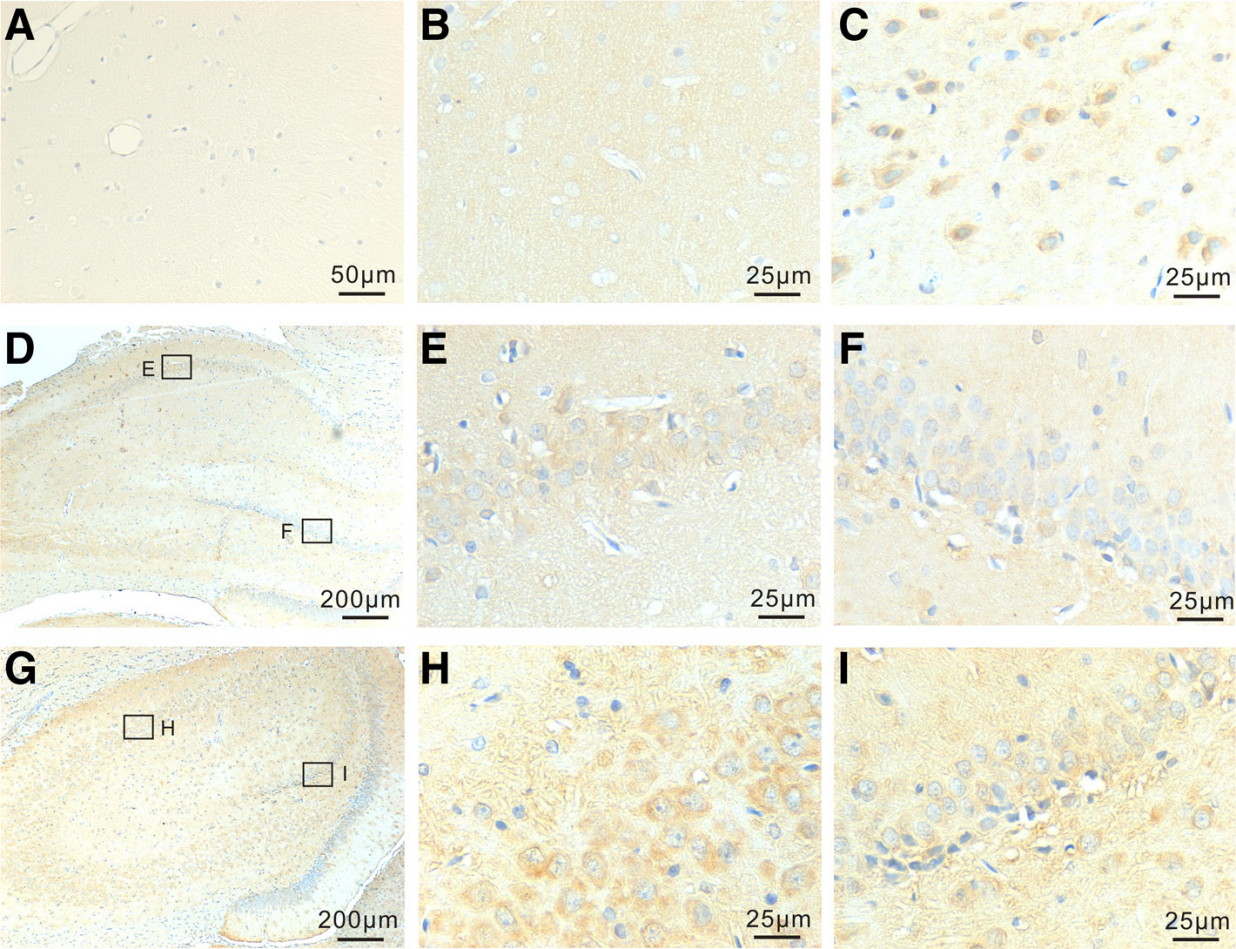
Immunohistochemical labeling of AhR in neurons of normal brains (**b**, **d**, **e**, and **f**) and brains 3 days post-TBI. **a** The specificity of AhR antibody was demonstrated by staining without the primary antibody and no IR was seen. **b**, **c** Examples of coronal sections of the cortex from normal brains (**b**) and brains 3 days after TBI (**c**). AhR expression (blue dots) was observed in the neurons of brain cortex in normal brains (**b**) and elevated three days post injury (**c**). **d**–**i** Examples of coronal sections of the AhR expression in hippocampus from normal brains (**d**–**f**) and brains 3 days after TBI (**g**–**i**). AhR expression was observed in neurons of pyramid cell layer of CA fields (**e** and **h**) and granular layer of dentate gyrus (**f** and **i**) in normal brains (**d**–**f**) and elevated in brains 3 days after TBI (**g**–**i**). The boxed areas indicate regions further observed under high-power magnification. The lower boxed areas represent the area of dentate gyrus and the upper boxed areas hippocampal CA fields

The expression of AhR in TBI and non-TBI brains was analyzed by immunohistochemistry. In our applied weight-drop model, cortex is the primary injury site. Moreover, the traumatic impact may influence areas like the hippocampus, resulting in complex cognitive problems. Therefore, the expression of AhR in cortex and hippocampus was mainly investigated. In in the cortex of normal rat brains, weak AhR immunoreactivity (IR) was observed, which mainly localized to neuron cells (Fig. 1a, b). Similarlly, weak IR of AhR was detected in hippocampus of normal rats, which was observed in the pyramid cell layer of CA fields (Fig. 1c, d) and granular layer of dentate gyrus (Fig. 1e).

After TBI, no visible change of AhR IR was detected in the contralateral hemisphere (Data not shown). In the injured (ipsilateral) sides, AhR expression in neuron cells in the cortex outside of lesion and hippocampus was upregulated since day 3. As shown in Fig. 1c, g, h and i, more AhR+ cells were observed and the IR of AhR became stronger as well compared to normal control at day 3 post injury. Interestingly, while an upregulation of AhR was observed following TBI, IR of AhR in neuron cells in the cortex and hippocampus was mainly localized to cytoplasm but not nucleus, indicating an inactivated state.

**Fig. 2:**
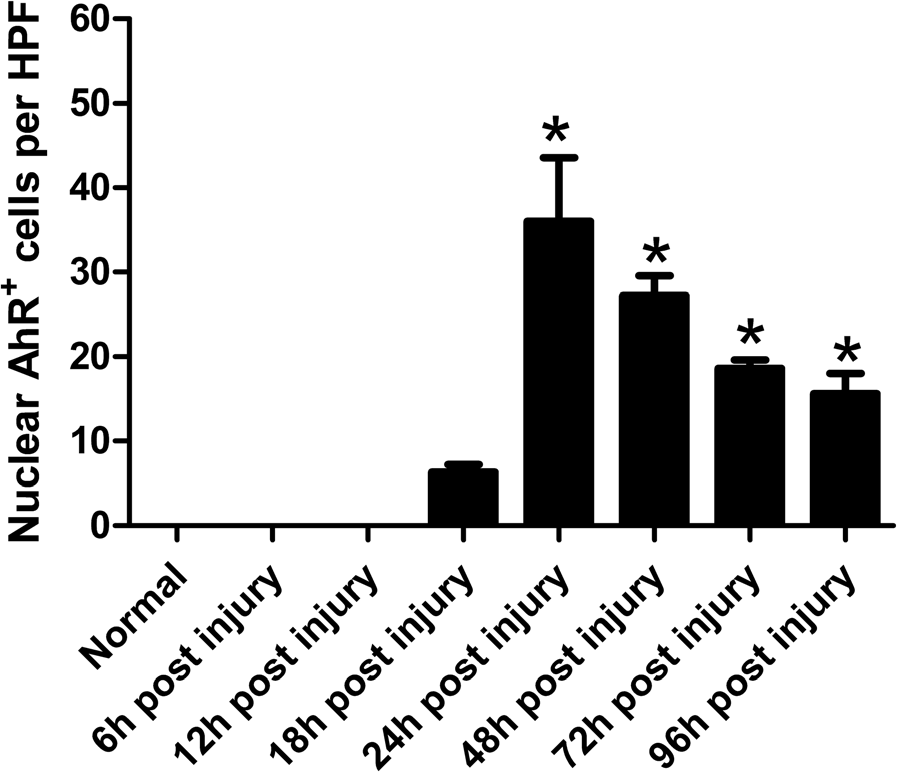
Time course of lesional AhR^+^ non-neuron cell accumulation in TBI. The numbers of parenchymal AhR^+^ non-neuron cells of every rat brain coronal section were counted in 8 HPFs. In each field, only positive cells with the nucleus at the focal plane were counted. Results were presented as arithmetic means of positive cells per HPF and standard errors of means (SEM). Statistical analysis was performed by one-way ANOVA followed by Dunnett’s Multiple Comparison test. *: *p* < 0.05 compared to normal control

**Fig. 3:**
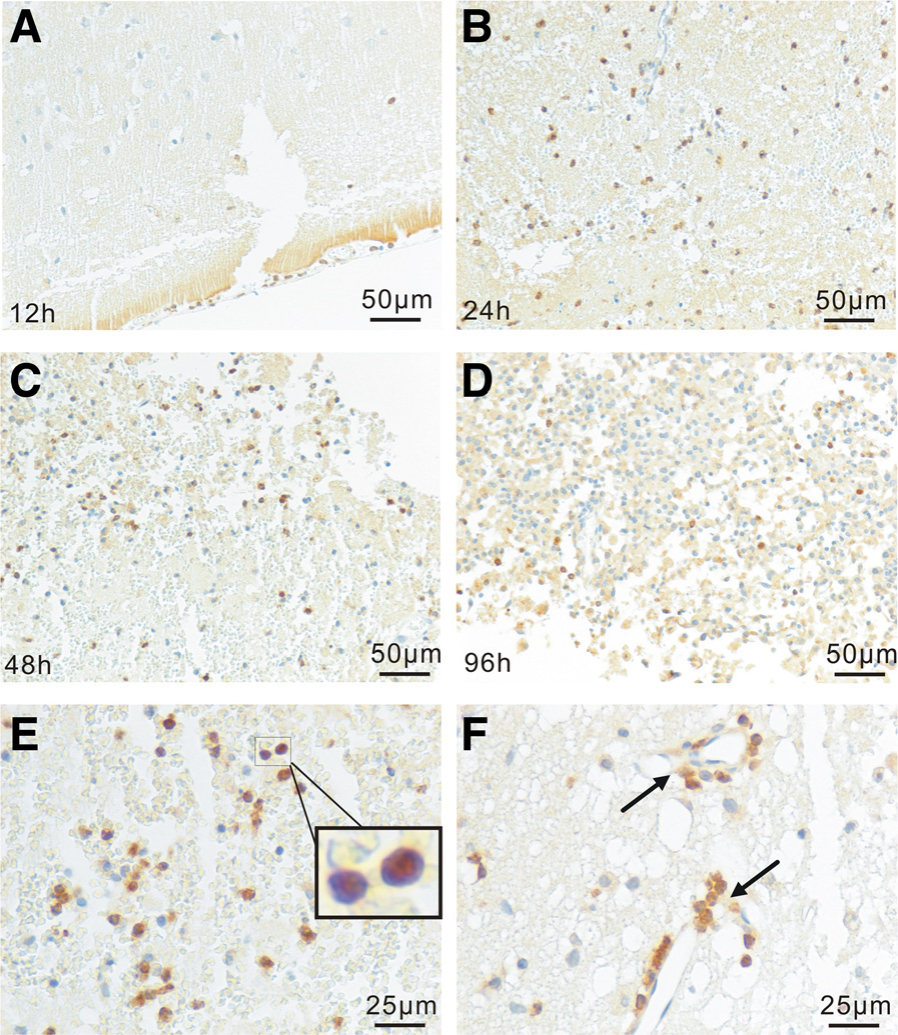
Accumulation of AhR^+^ non-neuronal cells in traumatic brains. In 12 h brains, AhR^+^ non-neuronal cells were rarely seen (**a**). Accumulation of AhR^+^ non-neuronal cells in the lesioned regions at day 1 (**b**), 2 (**c**) and 4 (**d**), respectively, after TBI. **e** Micrographs show that most AhR^+^ non-neuronal cells exhibited lymphocyte morphology with small cell body and cytoplasm (blue and brown dots). **f** Occasionally, the localization of AhR^+^ lymphocyte-like cells in the peri-vascular spaces corresponding to peri-vascular spaces was observed, indicating the blood origin. Furthermore, in AHR^+^ non-neuron cells, AhR was mainly localized to nucleus, suggesting an activated status (**e** and **f**)

However, the accumulation of non-neuron AhR^+^ cells was observed in the lesion areas. We then quantified the non-neuron AhR^+^ cells in lesional areas. After TBI, a slight increase of AhR^+^ non-neuron cells was observed to be confined to the lesion as early as 18 h post-injury (p.i.) (Fig. 2; 6.3 ± 1.5 per HPF, *p* > 0.05 compared to normal control). The maximal accumulation of AhR^+^ non-neuron cells in the lesional region was observed 24 h p.i. (36.0 ± 7.6 per HPF, *P* < 0.001; Fig. 2 and Fig. 3b). Thereafter the accumulation of AhR^+^ non-neuron cells decreased with time but still remained significantly higher than normal control (48 h: 27.2 ± 5.4 per HPF, *p* < 0.05; 96 h: 15.6 ± 5.4 per HPF, *p* < 0.05; Fig. 2 and Fig. 3c, d).

At the lesioned site, most of the AhR^+^ cells exhibited lymphocyte morphology with small cell body and cytoplasm (Fig. 3e). Occasionally, the localization of AhR^+^ lymphocyte-like cells at the peri-vascular spaces corresponding to peri-vascular spaces was observed, indicating the blood origin (Fig. 3f). More interestingly, in AhR^+^ non-neuron cells, AhR mainly localized in nucleus, suggesting an activated status (Fig. 3e, f).

**Fig. 4:**
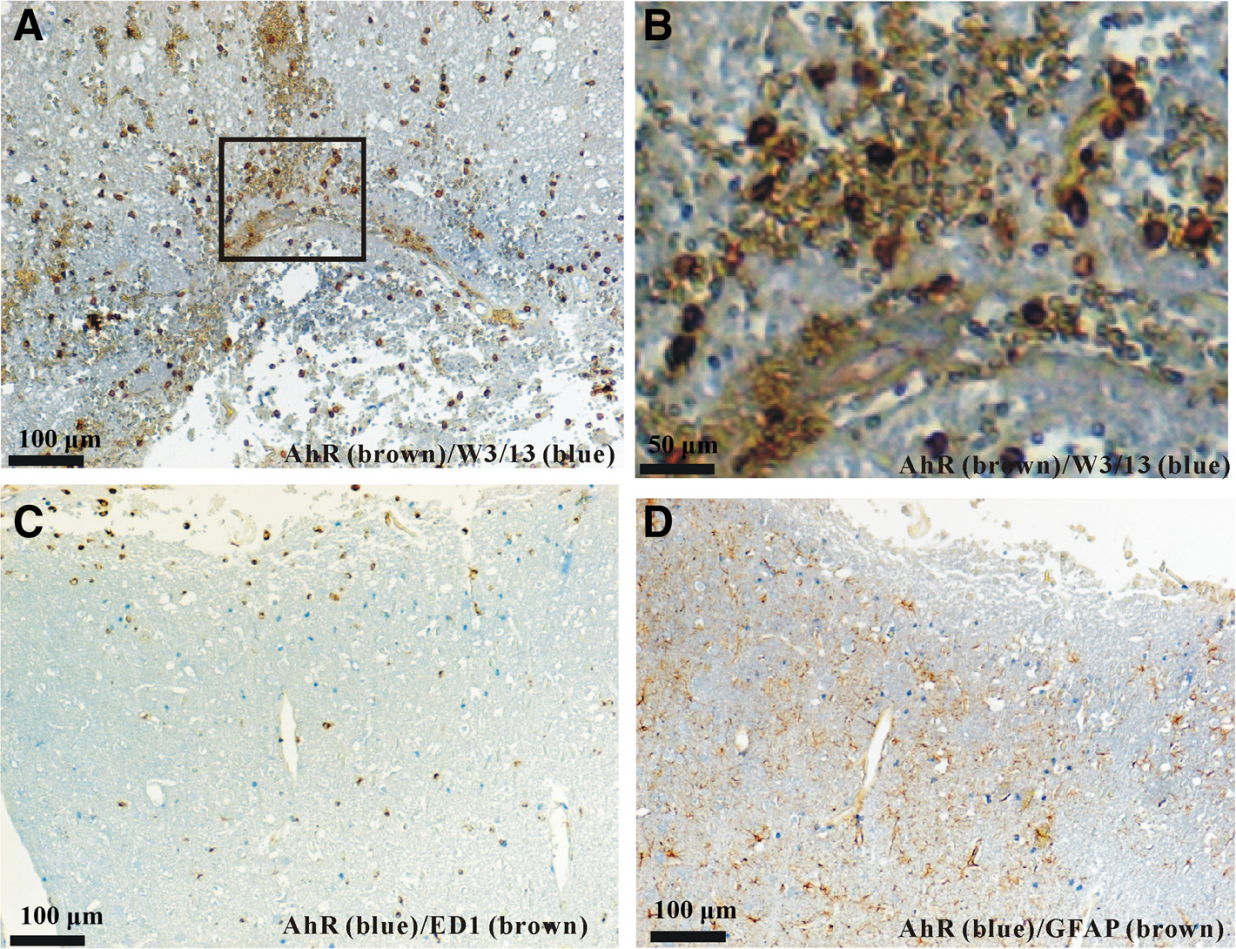
AhR double labeling in brain sections from day 1 after TBI. **a** Most AhR^+^ non-neuronal cells (brown) co-expressed W3/13 (blue). The boxed areas indicate the regions that were further observed under high-power magnification shown in (**b**). **c** and **d**: However, most AhR^+^ non-neuronal cells (blue) did not co-localize with ED1^+^ microglia (brown, **c**) or GFAP^+^ astrocytes (brown, **d**). Scale bar = 100 μm for (**a**, **c**) and **d**; 50 μm for (**b**)

To further identify the cellular sources of these non-neuron AhR^+^ cells in the lesions, we performed double-staining experiments with W3/13 antibody (recognized T lymphocytes), ED-1 antibody (recognized activated microglia/macrophages) and GFAP antibody (recognized astrocytes). As shown in Fig. 4a, b, most non-neuron AhR ^+^ cells were co-labeled with W3/13 (more than 90 %). ED-1^+^ microglia/macrophages and GFAP^+^ astrocytes very rarely co-expressed AhR near the lesional areas following TBI (Fig. 4c, d). Therefore T cells were the major cellular sources of non neuronal AhR in the lesion areas following TBI.

## Discussion

In this study, the expression of AhR in neurons of normal rat brain was mainly detected in the cortex and hippocampus in a low or moderate level, and occasionally found in the pyramid cell layer of CA fields, which was similar to previous report [15, 22]. In neurons, the expression of AhR increased following TBI but was mainly localized in the cytoplasm, indicating an inactivated state. However, due to the relative insensitivity of our applied method, a small amount of AhR may still exist in nucleus. Under pathological conditions, the over-expression of AhR was reported to induce neural differentiation [10] and AhR was also involved in controlling neuron cell cycle and apoptosis [11, 19].

The accumulation of AhR^+^ lymphocytes in lesional areas following TBI was observed in our study. Normally, T cells cannot enter the CNS. However, under pathological conditions, such as activated by CNS-derived auto-antigen and increased blood-brain barrier permeability, T cells can infiltrate into CNS parenchyma and exert multi-functions [23]. Clausen et al. reported that T lymphocytes (both CD4^+^and CD8^+^ cells) were detected in the lesional cortex at 12 h after TBI [3]. The reported infiltration, which is similar to our observation, peaked at 24 h and then slightly decreased.

Functions of T cells in brain injury are complicated and conflicting observations have been reported by different groups. Autoreactive Th1 cells by vaccination were reported to improve functional recovery after closed head injury [24]. Moreover, passive transferred autoreactive Th2 cells also accelerate recovery in spinal cord contusion [25]. However, immediate post-lesional transfer of MBP-specific T cells exacerbated lesion pathology in spinal cord contusion [26]. Furthermore, depletion of regulatory T cells (Treg) by CD25 antibody, reported by Liesz et al., profoundly increased delayed brain damage and deteriorated functional outcome in acute experimental stroke [27]. CD8^+^ T cells were less studied in injury models, but they are proved to infiltrate the lesion after TBI and may also play a part in the pathological process following TBI [3].

Currently, AhR can be detected in naïve T cells [28] and its expression was reported in the Th17 and Treg subsets [29] but not in Th1 or Th2 subsets [28]. Negishi et al. reported a modulation of the naïve Th cell differentiation into Th1/Th2 cells toward Th1 dominance under the activation of AhR treated by representative AhR ligands in vitro [28]. Moreover, 2,3,7,8-tetrachlorodibenzo-p-dioxin (TCDD), the exogenous AhR ligand, promoted Treg differentiation [18], while tryptophan photoproducts 6-formylindolo-[3,2-b]-carbazole (FICZ) would promote Th17 cell differentiation [18, 19]. AhR can also be expressed in CD8^+^ T cells and promote CD8^+^ Treg cell differentiation under TCDD treatment [30]. In our investigation, accumulation of AhR^+^ T lymphocytes was observed following TBI. Though the exact function of AhR in TBI still remains unclear, AhR might modulate the differentiation of T lymphocytes to influence the balance of immune response and contribute to the outcome of TBI [28, 29].

## Conclusions

Taken together, we have shown an early accumulation of activated AhR^+^ T cells in TBI. AhR expression might play a role in the degeneration/regeneration processes following TBI by modulating the differentiation of T lymphocytes. Our results warrant future studies of the AhR function in brain trauma.

## Abbreviations

AhR, Aryl hydrocarbon receptor; bHLH/PAS, basic helix-loop-helix/per-ARNT-Sim; DAB, diaminobenzidine; EAA, excitatory amino acid; FICZ, 6-formylindolo-[3,2-b]-carbazole; HE, Harris Hematoxylin and Eosin; HPFs, high-power fields; p.i., post-injury; SEM, standard errors of means; TBI, traumatic brain injury
